# Supplementary material for: Repeat DNA-PAINT suppresses background and non-specific signals in optical nanoscopy
Source: Nat Commun. 2021 Jan 21;12:501. doi: 10.1038/s41467-020-20686-z (PMC7820506; doi:10.1038/s41467-020-20686-z)
Supplement: Supplementary file 2 — Reporting Summary [file 41467_2020_20686_MOESM2_ESM.pdf]

nature research

Corresponding author(s): Christian Soeller

Last updated by author(s): 25/11/20

Reporting Summary

Nature Research wishes to improve the reproducibility of the work that we publish. This form provides structure for consistency and transparency in reporting. For further information on Nature Research policies, see our Editorial Policies and the Editorial Policy Checklist.

Statistics

For all statistical analyses, confirm that the following items are present in the figure legend, table legend, main text, or Methods section.

n/a	Confirmed	
☐	☒	The exact sample size (*n*) for each experimental group/condition, given as a discrete number and unit of measurement
☐	☒	A statement on whether measurements were taken from distinct samples or whether the same sample was measured repeatedly
☒	☐	The statistical test(s) used AND whether they are one- or two-sided<br>*Only common tests should be described solely by name; describe more complex techniques in the Methods section.*
☒	☐	A description of all covariates tested
☒	☐	A description of any assumptions or corrections, such as tests of normality and adjustment for multiple comparisons
☐	☒	A full description of the statistical parameters including central tendency (e.g. means) or other basic estimates (e.g. regression coefficient) AND variation (e.g. standard deviation) or associated estimates of uncertainty (e.g. confidence intervals)
☒	☐	For null hypothesis testing, the test statistic (e.g. *F*, *t*, *r*) with confidence intervals, effect sizes, degrees of freedom and *P* value noted<br>*Give P values as exact values whenever suitable.*
☒	☐	For Bayesian analysis, information on the choice of priors and Markov chain Monte Carlo settings
☒	☐	For hierarchical and complex designs, identification of the appropriate level for tests and full reporting of outcomes
☒	☐	Estimates of effect sizes (e.g. Cohen's *d*, Pearson's *r*), indicating how they were calculated

Our web collection on statistics for biologists contains articles on many of the points above.

Software and code

Policy information about availability of computer code

Data collection	Experimental data was collected using the Python software package PyME (Python Microscopy Environment), which is available at https://github.com/python-microscopy/python-microscopy (version 20.11.25). Simulations were carried out using a modified version of the oxDNA package available at https://github.com/WillTKaufhold1/oxDNA-no-self-bonds.
Data analysis	Data was analyzed using the Python software package PyME (Python Microscopy Environment), which is available at https://github.com/python-microscopy/python-microscopy (version 20.11.25) and also using the the set of plugins available at https://github.com/csoeller/PYME-extra (version 20.11.20).

For manuscripts utilizing custom algorithms or software that are central to the research but not yet described in published literature, software must be made available to editors and reviewers. We strongly encourage code deposition in a community repository (e.g. GitHub). See the Nature Research guidelines for submitting code & software for further information.

Data

Policy information about availability of data

All manuscripts must include a data availability statement. This statement should provide the following information, where applicable:

- Accession codes, unique identifiers, or web links for publicly available datasets
- A list of figures that have associated raw data
- A description of any restrictions on data availability

All data supporting this study are available upon reasonable request. Source data for Figures 1b, 1d, 2b, 2d, 4 & 5e have been provided with this paper.

Field-specific reporting

Please select the one below that is the best fit for your research. If you are not sure, read the appropriate sections before making your selection.

☒ Life sciences ☐ Behavioural & social sciences ☐ Ecological, evolutionary & environmental sciences

For a reference copy of the document with all sections, see nature.com/documents/nr-reporting-summary-flat.pdf

Life sciences study design

All studies must disclose on these points even when the disclosure is negative.

Sample size	Sample sizes were chosen to be at least 3 individual imaging chambers with labeled tissue, imaged on different days, as technical replicates for single molecules measurements testing the application of xRD strands. Replicates consisted of >100 k events which could be used to generate statistically significant measurements. Origami sample sizes were driven by the number of origami tiles. N = 10 tiles were used to measure the FWHM and the distance between individual points as a single origami provided multiple distinguishable sites. Event rates determined from synthetic samples utilized >150 events per origami tile which were used to estimate mean event rates.
Data exclusions	Localization events were filtered inside PyME based on a series of filters rejecting poorly localized single molecule events (typically for localisation error > 20 nm). This data was therefore excluded when rendering the super-resolution images.
Replication	Experiments where 1xRD strands were replaced with 10xRD strands in the same experiment were repeated n = 7 times in biological samples. Successful repetitions were determined by event rate monitoring. As 1xRD was introduced to the sample containing a compatible imager, the event rates increased, sampling the expected structure as identified by first having viewed the widefield image from the dye modified docking strand. When the removal sequence was added event rates decreased back down to background levels and after attaching and washing excess 10xRD, event rates were comparable to those measured at the higher imager concentration used for 1xRD imaging. Origami experiments showing similar results were repeated n = 7 times and were deemed to be successful based on observing single molecule events matching the designed structure. All replication attempts and imaging with xRD strands were successful.
Randomization	Randomization was not required to demonstrate that the repeat motif DNA-PAINT technique worked. Without the xRD strand no image of the underlying labeled structure could be probed when only imager complementary to the xRD strand was present. Only once the anchor strand was functionalized with xRD could the sample be imaged with this imager.
Blinding	Blinding was not done in this study. The experimenter needed to know approximately what imager concentration to add to samples. Being an order of magnitude different between 1xRD and 10xRD the effects would have been obvious.

Reporting for specific materials, systems and methods

We require information from authors about some types of materials, experimental systems and methods used in many studies. Here, indicate whether each material, system or method listed is relevant to your study. If you are not sure if a list item applies to your research, read the appropriate section before selecting a response.

Materials & experimental systems

n/a	Involved in the study	
☐	☒	Antibodies
☒	☐	Eukaryotic cell lines
☒	☐	Palaeontology and archaeology
☒	☐	Animals and other organisms
☒	☐	Human research participants
☒	☐	Clinical data
☒	☐	Dual use research of concern

Methods

n/a	Involved in the study	
☒	☐	ChIP-seq
☒	☐	Flow cytometry
☒	☐	MRI-based neuroimaging

Antibodies

Antibodies used	1. Ryanodine Receptor Monoclonal Antibody (C3-33), Mouse, (ThermoFisher MA3-916).<br>2. AffiniPure Goat Anti-Mouse secondary antibody (affinity purified, Jackson ImmunoResearch, 115-005-003).<br>3. Anti-alpha-actinin (Sarcomeric) monoclonal antibody (EA-53) Mouse, (Sigma, A7732).
Validation	MA3-916 has been well characterized for its high specificity and has been established as a consistently reliable marker for diffraction limited (Soeller et al, Proceedings of the National Academy of Sciences of the United States of America, 2007), (Jayasinghe et al, Biophysical Journal, 2009) and super-resolution microscopies (Baddeley, Proceedings of the National Academy of Sciences of the United States of America, 2009), (Hou et al, Journal of molecular and cellular cardiology, 2015), (Jayasinghe et al, Cell reports, 2018) of RyR2.<br>A7732 (for alpha-actinin) has previously been used to study Z-lines in cardiac samples imaged with confocal microscopy, (Soeller et al, Experimental Physiology, 2009).
